# Mesenteric lymphangioma in childhood: a case report and narrative literature review

**DOI:** 10.3389/fonc.2025.1541445

**Published:** 2025-05-09

**Authors:** Jin Li, Le Luo, Yong Liu, Wenlan Li, Xin Wei

**Affiliations:** ^1^ Department of Ultrasound, Deyang People’s Hospital, Deyang, China; ^2^ Medical Records Statistics Management Section, Deyang People’s Hospital, Deyang, China; ^3^ Department of Pediatric Surgery, Deyang People’s Hospital, Deyang, China

**Keywords:** children, abdominal cystic lymphangioma, abdominal pain, ultrasonography, laparoscopic surgical procedure, case report

## Abstract

Lymphangioma is an uncommon benign neoplasm of the lymphatic system, predominantly observed in children and infants and infrequently in adults. Mesenteric lymphangioma is very rare. This article reports on the case of a 4-year-old female who was admitted to the hospital due to abdominal pain. Ultrasonography identified a cystic mass in the abdominal cavity, diagnosed as mesenteric lymphangioma, for which surgical intervention was advised. The patient underwent laparoscopic lesion resection with small bowel resection and anastomosis under general anesthesia. Pathological examination confirmed the diagnosis of mesenteric lymphangioma. In children, it frequently presents as an acute abdomen. Our comprehensive literature analysis strongly suggests that treatment decisions for pediatric mesenteric lymphangioma need to be guided by a careful assessment of individual patient presentations. Although exploratory laparotomy with tumor resection and involved bowel segment removal remains the gold-standard treatment, the advent of laparoscopic techniques and sclerotherapy has facilitated the evolution of personalized therapeutic strategies, potentially reducing dependence on conventional surgical approaches in the future.

## Introduction

Mesenteric lymphangioma is a rare benign neoplasm of the lymphatic system, accounting for less than 1% of all lymphangiomas (1). Most studies consider lymphangiomas to be congenital developmental malformations caused by abnormalities of the embryonic lymphatic system (2). In general, mesenteric lymphangiomas appear as a painless tumor that grows gradually. It is frequently only identified when complications arise, which can result in possible fatal disorders such as intestinal obstruction, volvulus, intracystic infection, hemorrhage, urinary tract obstruction, etc. because it lacks specific clinical symptoms and indicators (3, 4). Early detection, precise diagnosis, and timely surgical intervention are important factors for avoiding serious consequences. The non-specific clinical symptoms of mesenteric lymphangioma make preoperative diagnosis challenging. The most useful imaging techniques for assessing abdominal cysts are ultrasound, CT, and MRI; however, postoperative pathological testing is still necessary for a conclusive diagnosis (5, 6). The diagnosis and management of a child’s mesenteric lymphangioma are discussed in this article. Furthermore, this study examined the data that is currently accessible on patient mesenteric lymphangioma.

## Case presentation

**Figure 1:**
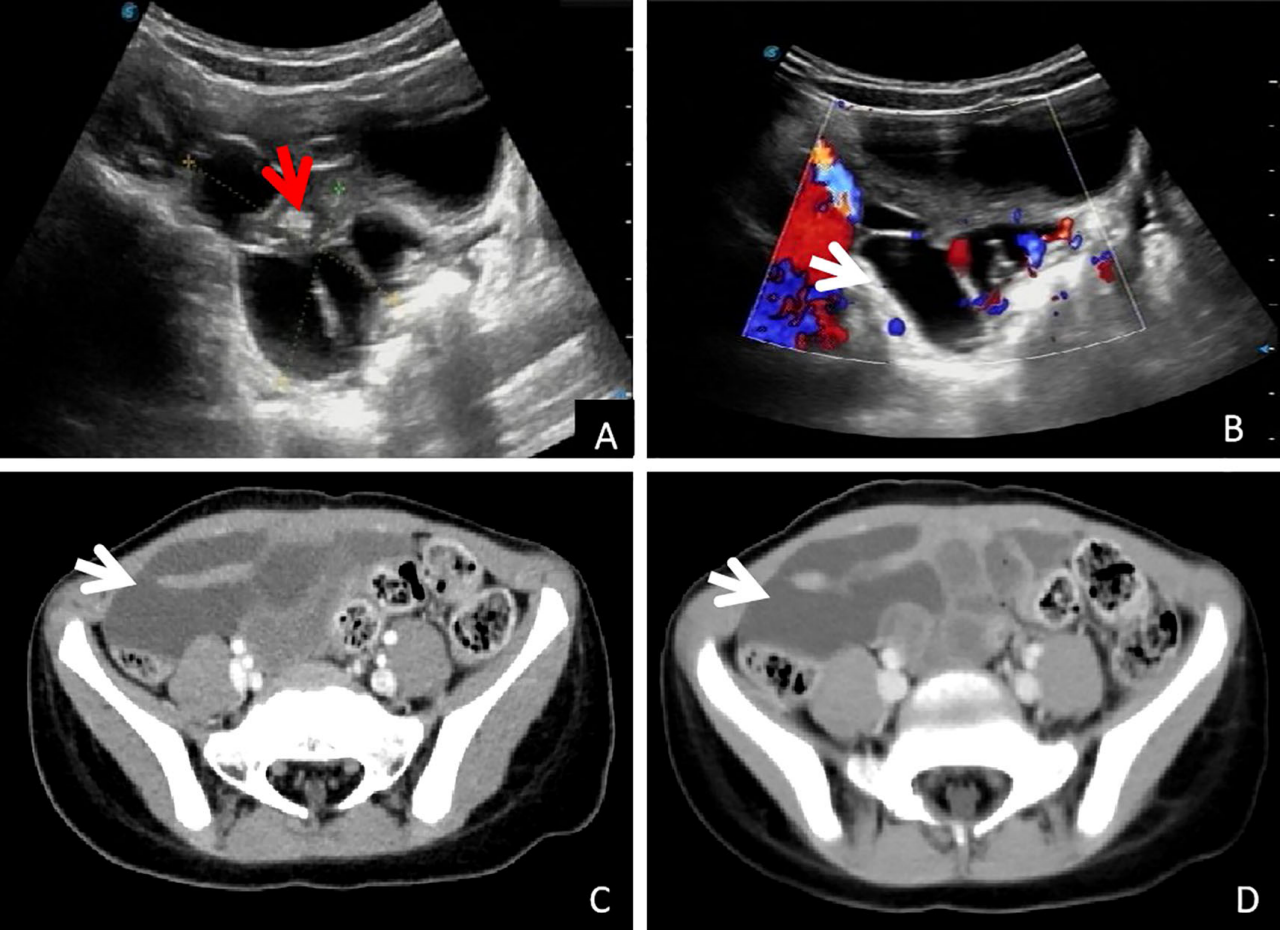
Imaging examination: **(A, B)**: Conventional ultrasound examination showed pelvic cystic occupying, visible separation (white arrow) and wrapped small intestine (red arrow), and blood flow signals with stars on the CDFI septum (white arrow). **(C, D)**: Abdominal enhanced CT showed cystic mass in the right pelvic cavity, and no enhancement was observed in arterial and venous phases (white arrow).

A 4-year-old girl was admitted to our hospital 5 months ago with a complaint of abdominal pain, and an abdominal mass was detected on ultrasound. Surgical intervention was recommended due to a suspected lymphangioma. The family opted for close observation and elective surgery, resulting in her discharge. One week before this admission, the child showed persistent abdominal pain, which was manageable and not associated with chills, fever, or abdominal distension. The child returned to our hospital for further evaluation. A second color Doppler ultrasound revealed a cystic mass measuring 4.9 × 5.2 × 3.6 cm in the lower abdominal cavity, characterized by an irregular shape with multiple strip-like septa and no significant peristalsis. There was no apparent change from the first examination. The findings were suggestive of mesenteric lymphangioma (**Figures 1A, B**).

**Figure 2:**
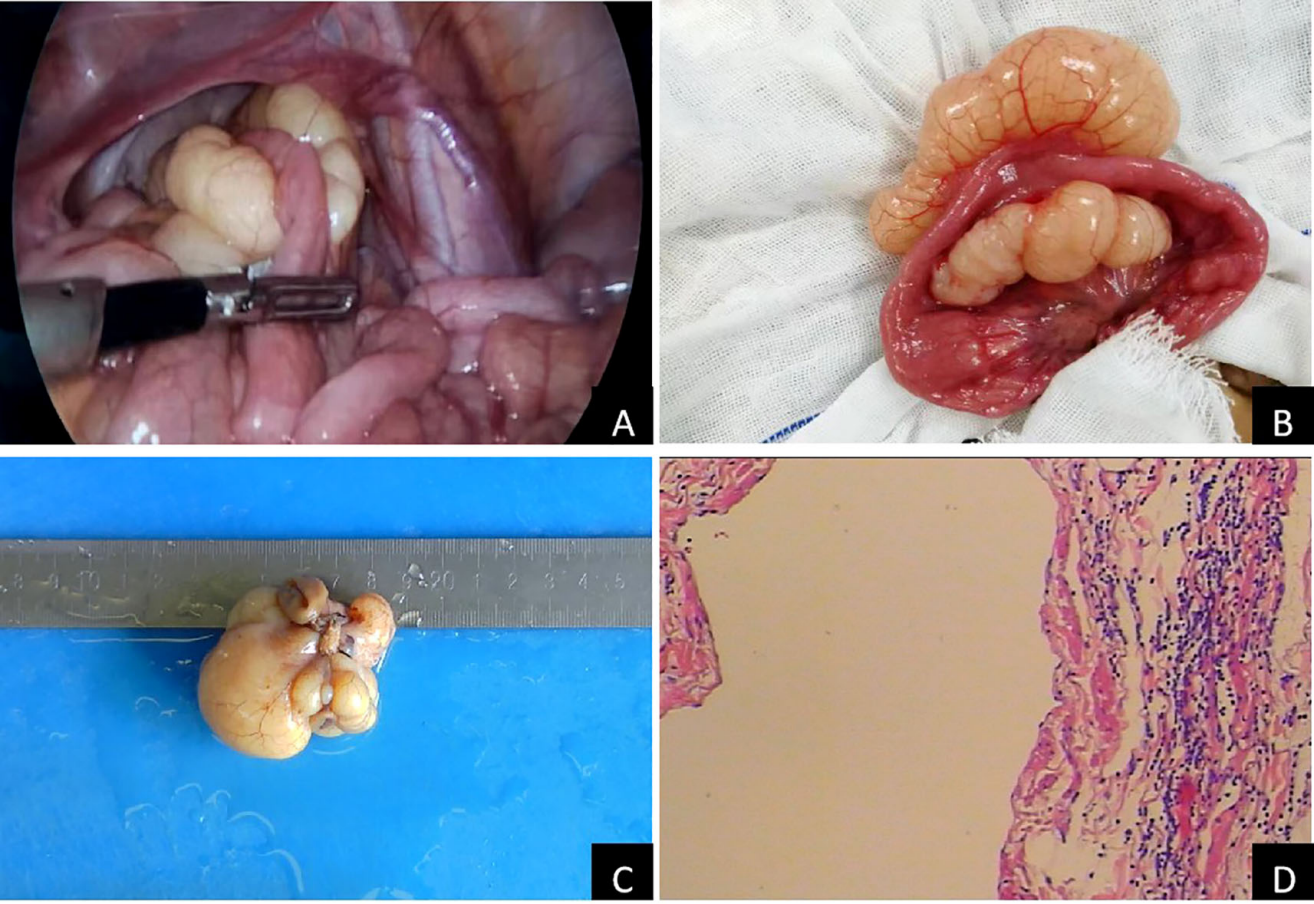
Pathological characteristics of laparoscopic surgery and mass: **(A)**: laparoscopic surgery; **(B)**: Lesions found during the operation; **(C)**: gross specimen of mass; **(D)**: Pathological examination of mass (H&E 100×).

Abdominal computed tomography (CT) further confirmed the presence of a cystic mass in the right lower abdominal cavity, with no enhancement observed in either the arterial or venous phases (**Figures 1C, D**, arrows). The child eventually underwent laparoscopic resection of the mesenteric lesion, accompanied by small bowel resection and anastomosis, under general anesthesia. A yellowish, irregularly shaped neoplasm, approximately 6 × 5 × 4 cm in size, was identified in the mesentery of the small intestine, located 100 cm from the ileocecal junction during the intraoperative assessment. The mass was solid, soft in consistency, and did not communicate with the intestinal lumen, completely encasing a segment of the small intestine approximately 5 cm in length (**Figures 2A, B**). The pathological examination confirmed the diagnosis of mesenteric lymphangioma (**Figures 2C, D**). At the 6-month follow-up, the child displayed no indications of abdominal pain, distension, or other discomforts. The follow-up abdominal ultrasound revealed no significant abnormalities, and the patient’s recovery was considered satisfactory.

## Discussion

Lymphangiomas are malformations of the lymphatic system, characterized by endothelial cell proliferation in thin-walled lymphatic channels and surrounding connective tissue. They are not true tumors but rather congenital developmental defects (2, 4). Most lymphangiomas occur in regions with abundant loose connective tissue, such as the neck, axilla, and groin, with rare abdominal and mediastinal involvement. Mesenteric lymphangiomas account for less than 1% of all cases (1). Lymphangiomas are believed to result from congenital developmental defects of the lymphatic system (3). However, certain studies indicate that abdominal surgery, trauma, radiotherapy, or inflammation may impair lymphoid tissue, forming lymphangiomas (1, 7). The case described aligns with the concept of congenital lymphatic malformation, given the patient was a preschool-aged child with no history of trauma or infection.

In this study, a Pubmed literature search from January 1930 to February 2025 was carried out while employing the terms “mesentery” and “lymphangioma”. Each abstract was carefully examined by two independent authors (FP and IL), with the inclusion criteria being (I) mesenteric CL, (II) English manuscript, (III) availability of complete text, and (IV) subjects less than 18 years of age. A literature study revealed a total of 69 cases of pediatric mesenteric CL. **Table 1** summarizes the characteristics of pediatric mesenteric CL cases reported in the literature. Most of the patients were generally male, with a mean age of 66.22 (± 5.04) years and a mean cyst size of 13.60 (± 6.34) cm. The most common clinical symptoms were abdominal pain (72.46%, 50 cases), followed by abdominal distension, vomiting, constipation, and fever. The most common sign was abdominal mass palpation (46.38%, 32 cases), followed by abdominal tenderness and distension. Ultrasound examinations accounted for 56.52% of the initial diagnosis (39 cases), with CT examinations coming in second. The most common symptom of acute abdomen in 46.38% (32 cases) of patients was volvulus (39.13%, 27 cases). Intestinal obstruction, infection, urinary obstruction, and cachexia were the following most common symptoms. The lesions were mainly located in small mesentery (82.61%, 57 cases), followed by mesocolon (10.14%, 7 cases). Multilocular cystic lesions were the most prevalent morphology (78.26%, 54 cases), followed by unilocular cystic lesions (11.59%, 8 cases). Surgery was performed on 98.55% of the patients, with the majority undergoing open resection (94.12%, 64 cases) and only a small number undergoing laparoscopic resection (5.88%, 4 cases). The outcomes were favorable, with only one death, complication, and recurrence.

**Table 1:** Characteristics of pediatric cases of mesenteric lymphangioma reported in the literature.

Characteristics	n=69
**Gender (male)**	45 (65.20)
**Age**	6.22 (±5.04)
**Tumor size (cm)**	13.6 (±6.34)
Clinical manifestations	
None	8 (11.59)
Abdominal pain	50 (72.46)
abdominal distension	21 (30.43)
Vomiting	27 (39.13)
Constipation	11 (15.94)
Fever	11 (15.94)
respiratory distress	2 (2.9)
Loss of appetite	5 (7.25)
anuria	2 (2.90)
Gastrointestinal bleeding	1 (1.45)
Diarrhea	1 (1.45)
Physical examination	
None	12 (17.39)
Abdominal mass	32 (46.38)
Tenderness on palpation	25 (36.23)
abdominal protuberant	20 (28.99)
Muscle tension	3 (4.35)
Unknown	2 (2.90)
Initial Imaging Studies	
US	39 (56.52)
Abdominal CT	13 (18.84)
X-ray	10 (14.49)
MRI	1 (1.45)
Other	6 (8.70)
**Complications (Y)**	43 (62.32)
None	26 (37.68)
Acute abdomen (Y)	32 (46.38)
intestinal volvulus	27 (39.13)
intestinal obstruction	15 (21.74)
infected	10 (14.49)
obstructive uropathy	4 (5.80)
Cachexia	4 (5.80)
Other	16 (23.19)
Lesion location	
small intestinal	57 (82.61)
jejunal	26 (45.61)
ileal	10 (17.54)
colon	7 (10.14)
small intestinal and colon	2 (2.90)
Unknown	3 (4.35)
Lesion morphology	
multiloculated fused cystic lesions	54 (78.26)
the twin cystic lesions	3 (4.35)
a large, thin-walled cystic mass	8 (11.59)
semi-solid cystic mass	2 (2.90)
Unknown	2 (2.90)
Treatment	
Conservative	1 (1.45)
Surgery	68 (98.55)
Open	64 (94.12)
Laparoscopy	4 (5.88)
**Postoperative complications**	2 (2.90)
**Recurrence**	1 (1.45)

Initial Imaging Studies: Imaging modality first identified; Complications: Complications occurring before treatment; Lesion morphology: Imaging revealed the internal structure of the lesion; Treatment: Methods of treatment; US: Ultrasound examination; Open: Open surgery; Vided as number of cases (%) or mean values (±standard deviations).

In summary, in line with previous studies (2, 6, 8), the most common clinical presentation of mesenteric lymphangiocele in children is abdominal pain, with or without a palpable abdominal mass. Clinical symptoms of pediatric mesenteric lymphangioma include abdominal distension, constipation, fever, weight loss, and peritonitis, and are frequently associated with the size, location, and complications of the tumor (3). A large percentage of children present to the hospital with acute abdominal pain or diverse digestive disorders. Intestinal volvulus is a prevalent complication, succeeded by intestinal obstruction, infection, urinary tract obstruction, cachexia, bleeding, as well as additional challenges, which may be deadly in severe cases (3, 8, 9). Therefore, early diagnosis and detection, rapid intervention, and surgical lesion removal are crucial to avoid complications and alleviate parents’ concerns. However, pediatric mesenteric lymphangioma presents with a variety of non-specific clinical symptoms.

Even with the introduction of high-performance imaging methods, preoperative diagnosis remains challenging. The best imaging techniques for assessing abdominal cysts include ultrasound, CT, and MRI. Accurate diagnosis is not always simple, though. Because of its simple, radiation-free, and reproducible features—which can make it extremely sensitive to show abnormalities—ultrasonography has emerged as the preferred imaging method (3, 5, 6, 9). As a result, understanding the ultrasonography features of mesenteric lymphangioma is crucial for enhancing diagnostic precision. The literature research indicated that the imaging characteristics of mesenteric lymphangioma were multilocular cystic masses. Grey-scale ultrasonography usually revealed an anechoic mass with clear borders, posterior enhancement, and apparent septation. Color Doppler imaging may reveal little blood flow within the septum. The location of the cystic lesion (mesentery, omentum, or retroperitoneum), the mass’s size, the cyst wall’s characteristics (thin or thick), the internal factors (unilocular or multilocular), and the contents (serous, chylous, or hemorrhagic) can all be revealed by computed tomography and MRI (3, 6, 10). This helps differentiate between intestinal duplication, ovarian cystic mass, choledochal cyst, ureter ectasia, urachal cyst, teratoma, and peritoneal fluid entrapment (3, 5, 11).

Despite being benign, mesenteric lymphangiomas can occasionally result in serious complications. The literature evaluation indicates that open surgery remains the recommended course of treatment for full excision of the tumor and affected bowel (2, 3, 6). However, compared with open surgery, laparoscopic surgery has the advantages of smaller incisions, less postoperative pain, shorter hospital stay, faster recovery, and reduced risk of complications such as intestinal obstruction and incision infection (5, 6, 12). Complete resection of local lymphangioma is optimal, but partial resection of multiple lesions may be required to reduce tumor volume and improve patient quality of life (3, 6, 11). Based on the earlier classification of mesenteric lymphangioma, Yan J et al. proposed improvements in terms of location and the degree of intestinal and mesentery involvement, dividing it into five types. The choice of suitable surgical methods can be guided by this classification (2, 3, 13). To achieve a positive outcome, multidisciplinary management and adjuvant therapies, like sclerotherapy, may be required for complex cases. In sclerotherapy, an experienced interventional radiologist, using ultrasound guidance, inserts a needle into the cystic cavity of the lymphangioma, positioning a guide wire and inserting a pigtail tube into the cystic cavity along the guide wire. The cystic fluid is suctioned through the pigtail tube, followed by injection of sclerosing agent into the cystic cavity. The sclerosing agent induces degeneration and necrosis of endothelial cells in the cyst wall, forming adhesions, to enable self-healing (14). Several studies have demonstrated the efficacy of sclerotherapy, which has since become the mainstay of treatment for large cystic lesions and is more effective than surgical resection and associated with a lower recurrence rate, as well as, more importantly, enabling effective preservation of important structures such as the intestine, nerves, arteries, and veins (14, 15). Although spontaneous regression of adult lymphangioma has been documented in a limited number of cases (16), certain investigators advocate for non-surgical management in asymptomatic patients with mesenteric lymphangioma (17). Our comprehensive literature analysis strongly suggests that treatment decisions for pediatric mesenteric lymphangioma need to be guided by a careful assessment of individual patient presentations. Surgical treatment should be performed in time for complications. Proactive intervention may not be warranted in asymptomatic cases or when complications are unlikely. Also in selected asymptomatic patients, non-surgical management or watchful waiting may be appropriate.

## Conclusions

The current study concluded that mesenteric lymphangioma is an uncommon benign condition with no specific clinical symptoms. In children, it frequently presents as an acute abdomen. Our comprehensive literature analysis strongly suggests that treatment decisions for pediatric mesenteric lymphangioma need to be guided by a careful assessment of individual patient presentations. Although exploratory laparotomy with tumor resection and involved bowel segment removal remains the gold-standard treatment, the advent of laparoscopic techniques and sclerotherapy has facilitated the evolution of personalized therapeutic strategies, potentially reducing dependence on conventional surgical approaches in the future.

## Data Availability

The raw data supporting the conclusions of this article will be made available by the authors, without undue reservation.
